# Constructing Localized Van Der Waals Gaps in Cubic‐Phase GeMnTe_2_ Thermoelectric Material

**DOI:** 10.1002/advs.202517830

**Published:** 2025-11-03

**Authors:** Mingrui Zhang, Lingling Wei, Tingting Yang, Weishuai Wang, Fudong Zhang, Mengqi Li, Beiquan Jia, Yalin Shi, Zupei Yang, Rafal E. Dunin‐Borkowski, Lei Jin, Di Wu

**Affiliations:** ^1^ Key Laboratory for Macromolecular Science of Shaanxi Province and Shaanxi Key Laboratory for Advanced Energy Devices School of Materials Science and Engineering Shaanxi Normal University Xi'an 710119 China; ^2^ School of Chemistry and Chemical Engineering Shaanxi Normal University Xi'an 710119 China; ^3^ Ernst Ruska‐Centre for Microscopy and Spectroscopy with Electrons Forschungszentrum Jülich GmbH 52425 Jülich Germany

**Keywords:** cubic GeMnTe_2_, synergistic optimization, thermoelectric materials, van der Waals gap

## Abstract

Cubic‐phase GeMnTe_2_ shows high potential to replace state‐of‐the‐art rhombohedral GeTe for medium temperature thermoelectric application owing to its lower cost. The high structural symmetry can also suppress phase transition during service and provides a superior platform for further band and microstructural engineering. Through Sb_2_Te_3_ alloying and Pb substitution, this study realizes a superior peak figure of merit of ≈1.5 at 773 K and a remarkable average figure of merit of ≈0.96 at 323–823 K. Sb_2_Te_3_ alloying successfully generates high‐density localized van der Waals (vdW) gaps which are able to scattering low‐frequency phonons effectively for reduced lattice conductivity; meanwhile, it also enlarges the valence band degeneracy for enhanced power factor. Pb substitution further reduces the hole concentration to an optimal level. The achievements in this work well reveal the efficacy of construing localized vdW gaps in improving matrix material's thermoelectric performance, thus might shed light on other cubic or pseudo‐cubic thermoelectric systems.

## Graphical abstract

Sb_2_Te_3_ alloying generates high‐density localized van der Waals gaps in cubic Ge_0.45_Mn_0.55_Te matrix, resulting in electron‐phonon decoupling and improved thermoelectric figure of merit *ZT*.

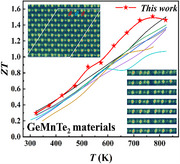

## 1 Introduction

Thermoelectric materials are a unique class of functional materials that can directly convert heat into electricity (Seebeck effect) or vice versa (Peltier effect).^[^ ^1^, ^2^, ^3^ ^]^ Due to their potential applications in power generation and solid‐state refrigeration,^[^ ^4^, ^5^, ^6^, ^7^ ^]^ thermoelectric materials attracted considerable attentions in recent decades. The performance of a thermoelectric material is primarily evaluated by the dimensionless figure of merit *ZT*, defined as *ZT = (S^2^σT)/κ_tot_*, where *S* is the Seebeck coefficient, *σ* is electrical conductivity, *T* is the absolute temperature in Kelvin, and *κ_tot_* is total thermal conductivity (comprising electronic part *κ_ele_* and lattice part *κ_lat_*). Naturally, a high *ZT* value requires a large power factor (*PF* = *S^2^σ*) and simultaneously a low κ_*tot*_;^[^ ^8^, ^9^, ^10^ ^]^ unfortunately, the interdependence of base parameters (*S*, *σ*, and κ_*tot*_) makes *ZT* enhancement quite challenging. Till now, strategies that have been used to enhance peak *ZT* can fall into one or the combination of the following points, including but not limited to modulating electronic bands, charge transfer engineering, optimizing carrier concentration, introducing point defects and/or nanostructure/dislocations, refining grains.^[^ ^11^, ^12^, ^13^, ^14^, ^15^, ^16^, ^17^, ^18^, ^19^, ^20^, ^21^, ^22^ ^]^

In addition to pursuing higher peak *ZT*, it is also of practical importance to achieve high average thermoelectric performance (i.e., average *ZT*) in a wider temperature range (in most cases at medium or high temperatures). Since the phase of a thermoelectric material during service is usually not its naturally stable form at low or room temperature, stabilization of this high‐performance (and high‐temperature) phase to low or even room temperature becomes a possible approach, as reported for SnSe,^[^ ^23^, ^24^, ^25^ ^]^ Cu_2‐_ *_x_* S,^[^ ^26^ ^]^ GeTe, *etc*. As for GeTe‐based materials with room temperature (RT) rhombohedral structure (space group: *R*3*m*), the *ZT* value can easily exceed 2.5 via band engineering and microstructure complication.^[^ ^27^, ^28^, ^29^ ^]^ However, a phase transition from rhombohedral to cubic structure at ≈700 K,^[^ ^30^ ^]^ is detrimental to achieving high average *ZT* as well as to maintaining good thermodynamic stability of thermoelectric module in the scenario of medium temperature service.^[^ ^31^, ^32^ ^]^ In contrast, recent studies show that reaction of rhombohedral GeTe and hexagonal MnTe can create a pseudo‐binary compound of GeMnTe_2_ (or written as Ge_0.5_Mn_0.5_Te), which exhibits a rock salt cubic structure at RT (space group: *Fm* $\bar{3}$ *m*) and *p*‐type conducting behavior.^[^ ^33^, ^34^ ^]^ The high‐symmetry crystal structure of GeMnTe_2_ as compared with rhombohedral GeTe results in a highly degenerated valence band structure, which is constructive to assure high *ZT*;^[^ ^35^, ^36^, ^37^ ^]^ Meanwhile, it eliminates the unwanted phase transition up to the working temperatures and also provides a superior platform for further microstructural engineering. In addition, the disordered distribution of Ge and Mn at cationic sites leads to intense phonon scattering rate thus quite low lattice thermal conductivity.^[^ ^33^, ^36^, ^38^ ^]^ Furthermore, because of the less comsumption of Ge in GeMnTe_2_, it shows a superior price advantage over the GeTe‐base materials. However, the extremely high intrinsic hole concentration (≈10^21^ cm^−3^) resulting from the large number of cation vacancies becomes the major obstruction of realizing decent thermoelectric performance in GeMnTe_2_.

In recent years, constructing localized van der Waals (vdW) planar defect structure in high‐symmetry lattice exhibits great potential in improving thermoelectric performance of matrix materials. Here, localized vdW is used in order to distinguish those that are intrinsically present (thus laterally wide and often periodically stacked) in layered vdW materials. It has been reported that Sb_2_Te_3_ alloying can successfully trigger localized vdW gaps in rhombohedral GeTe and cubic SnTe lattice.^[^ ^39^, ^40^, ^41^ ^]^ With nanoscale lateral dimensions and atomic‐scale vertical gaps, localized vdW gaps scatter phonons significantly and scatter carriers weakly compared to the laterally wide gaps,^[^ ^39^, ^42^, ^43^ ^]^ thus realizing the so‐call discriminately scattering of charge carriers and heat‐carrying phonons. It should be noted that higher symmetry (e.g., cubic) usually represents higher density of localized vdW gaps that can be introduced to the structure, which provides an extra parameter for microstructure engineering.

In this work, we first regulate the molar ratio of Ge/Mn in Ge_1‐_ *_x_* Mn*_x_* Te matrix in order to optimize the basal composition, then alloy Sb_2_Te_3_ into the basal material to construct localized vdW gaps like in GeTe;^[^ ^44^, ^45^, ^46^ ^]^ afterward, we optimize the hole concentration by doping Pb at Mn site. Ultimately, we achieve a notably improved thermoelectric performance in the composition of (Ge_0.45_Mn_0.4_Pb_0.15_Te)_0.8_(Sb_2/3_Te)_0.2_. The maximal *ZT* value reaches ≈1.5 at 773 K, and the average *ZT* value is ≈0.96 at the temperature range of 323–823 K. Meanwhile, the Vickers hardness *H_v_* is obviously enhanced from 180.7 for pristine GeMnTe_2_ to 230.7 for (Ge_0.45_Mn_0.4_Pb_0.15_Te)_0.8_(Sb_2/3_Te)_0.2_. This work reveals that GeMnTe_2_ could stand as a robust medium temperature thermoelectric material and that constructing localized vdW gaps could diversify the optimizing strategy for higher thermoelectric performance.

## 2 Results and Discussion

**Figure 1:**
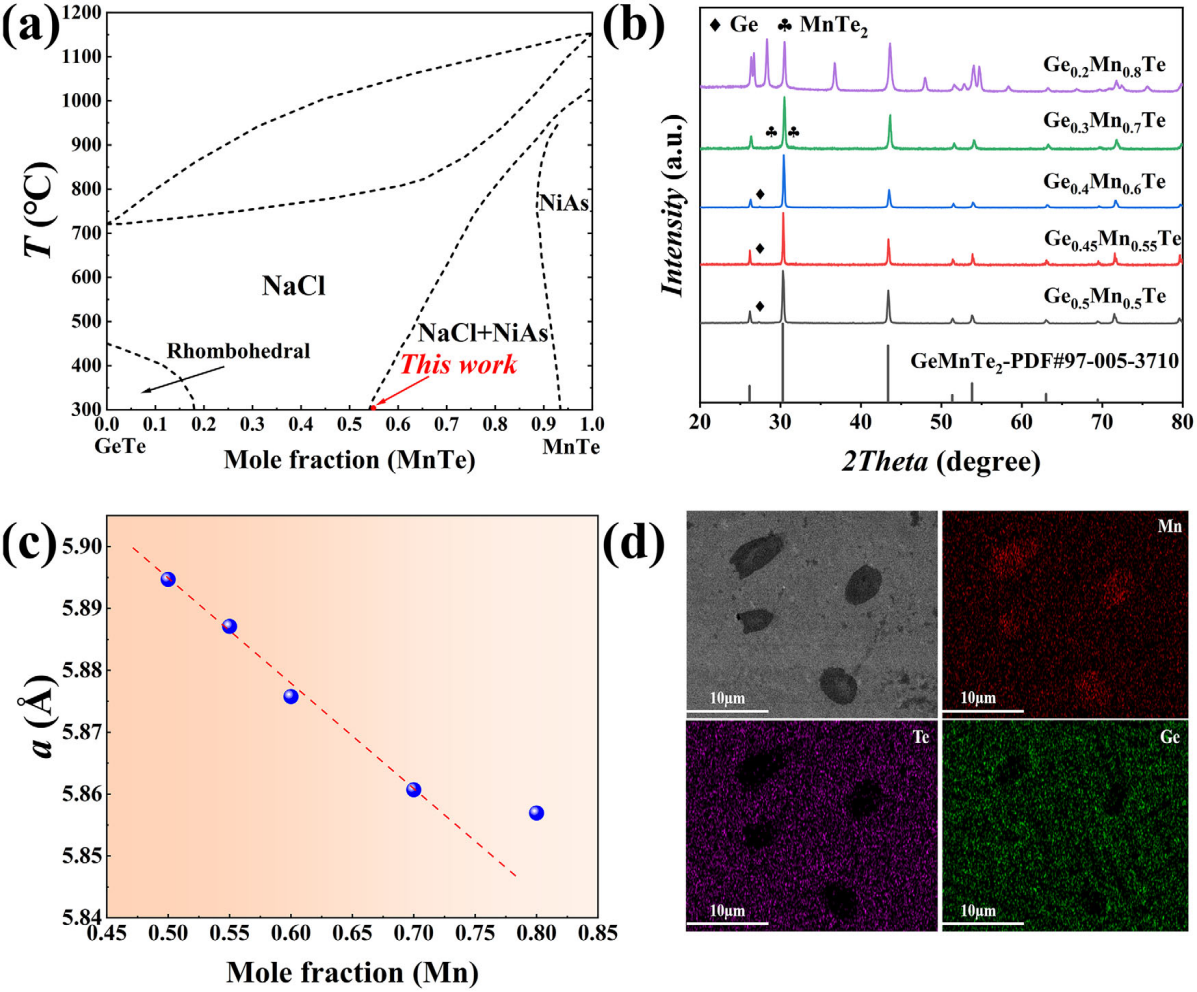
a) Replotted GeTe‐MnTe pseudo‐binary phase diagram based on Ref.;^[^ ^34^ ^]^ b) powder XRD patterns of Ge_1‐_ *_x_* Mn*_x_* Te (*x* = 0.5, 0.55, 0.6, 0.7, and 0.8), c) measured lattice parameters (of dominant cubic phase) as a function of Mn content *x*; d) SEM and corresponding EDS results of the Ge_0.2_Mn_0.8_Te sample.

**Figure** **1**a shows the replotted pseudo‐binary phase diagram of GeTe‐MnTe.^[^ ^34^ ^]^ It can be seen that Ge_1‐_ *_x_* Mn*_x_* Te can retain cubic structure when *x* lies between 0.18 to 0.55 and becomes a coexistence of cubic and hexagonal structures thereafter (*x* > 0.55). In literature, the composition of Ge_0.5_Mn_0.5_Te (*x* = 0.5) with a stable cubic structure at RT was used for thermoelectric studies.^[^ ^36^, ^38^, ^47^ ^]^ In this work, we first adjust the molar ratio of Ge/Mn in Ge_1‐_ *_x_* Mn*_x_* Te to optimize the basal material while maintaining the cubic structure. For the sake of cost, we increase *x* starting from *x* = 0.5. Figure 1b depicts the powder X‐ray diffraction (XRD) patterns of obtained Ge_1‐_ *_x_* Mn*_x_* Te (*x* = 0.5, 0.55, 0.6, 0.7, and 0.8) samples, and details of sample synthesis can be found in the Supporting Information (SI). All samples exhibit the cubic phase structure, and the measured lattice parameter decreases gradually as Mn content *x* increases, as shown in Figure 1c. This is mainly due to the smaller ionic radius of Mn^2+^ (0.67Å) as compared to Ge^2+^ (0.73 Å). Besides the dominant cubic phase, Ge precipitates are observed for *x* = 0.5, 0.55, and 0.6, and when *x* exceeds 0.7, diffraction peaks of MnTe_2_ appears. When *x* increases further to 0.8, a large number of heterogeneous Mn‐rich precipitations are observed in Figure 1d using scanning electron microscopy (SEM) and associated energy dispersive X‐ray spectroscopy (EDS), in consistence with XRD results.

**Figure 2:**
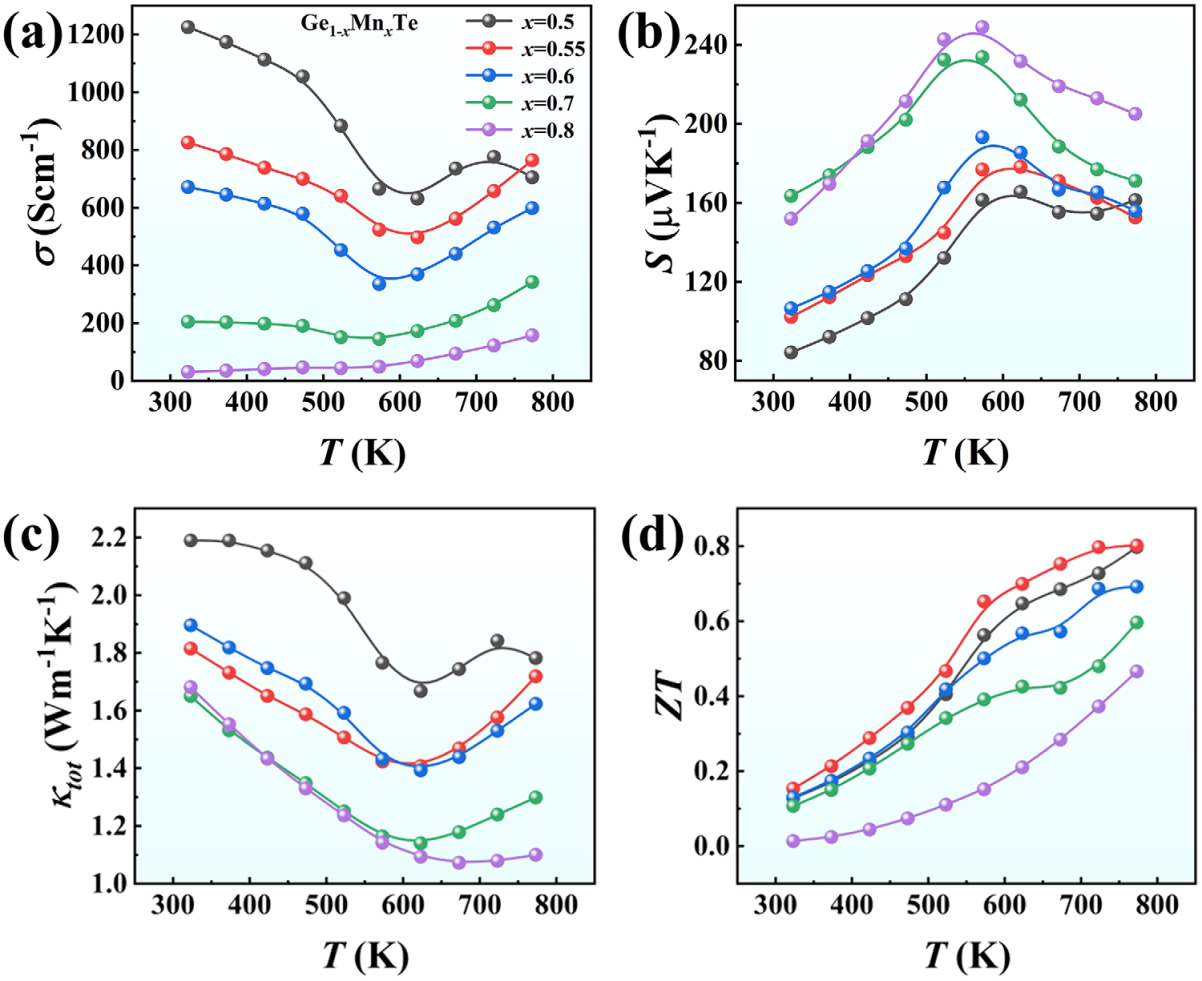
Temperature dependence of a) electrical conductivity *σ*, b) Seebeck coefficient *S*, c) thermal conductivity *κ_tot_*, and d) figure of merit *ZT* for Ge_1‐_ *_x_* Mn*_x_* Te (*x* = 0.5, 0.55, 0.6, 0.7, and 0.8).

The thermoelectric performance of Ge_1‐_ *_x_* Mn*_x_* Te (*x* = 0.5, 0.55, 0.6, 0.7, and 0.8) is shown in **Figure** **2**. The electrical conductivity *σ* initially decreases and subsequently increases with elevating temperature, as shown in Figure 2a, which might be related to the thermal excitation of minority charge carriers. Moreover, *σ* tends to decrease with increasing Mn content *x*, which is opposite to the behavior of Seebeck coefficient *S* as shown in Figure 2b. The *S* values of all these samples are positive, exhibiting a *p*‐type character with hole as the dominant charge carriers. Specifically, *S* at RT can rise significantly up to 163 µV K^−1^ in the sample with *x* = 0.7, as compared to only 84 µV K^−1^ for the control sample Ge_0.5_Mn_0.5_Te. The total thermal conductivity *κ_tot_* is reduced from 2.2 W m^−1^ K^−1^ for Ge_0.5_Mn_0.5_Te to 1.8 W m^−1^ K^−1^ for Ge_0.45_Mn_0.55_Te at RT, and the upturn of *κ_tot_* at high temperature is attributed to the bipolar effects (Figure 2c).^[^ ^48^, ^49^ ^]^ Eventually, the Ge_0.45_Mn_0.55_Te sample exhibits a comparable maximal *ZT* of ≈0.8 at 773 K with the control sample Ge_0.5_Mn_0.5_Te, but a much higher average *ZT* of ≈0.5 from 323 to 773 K (Figure 2d).

**Figure 3:**
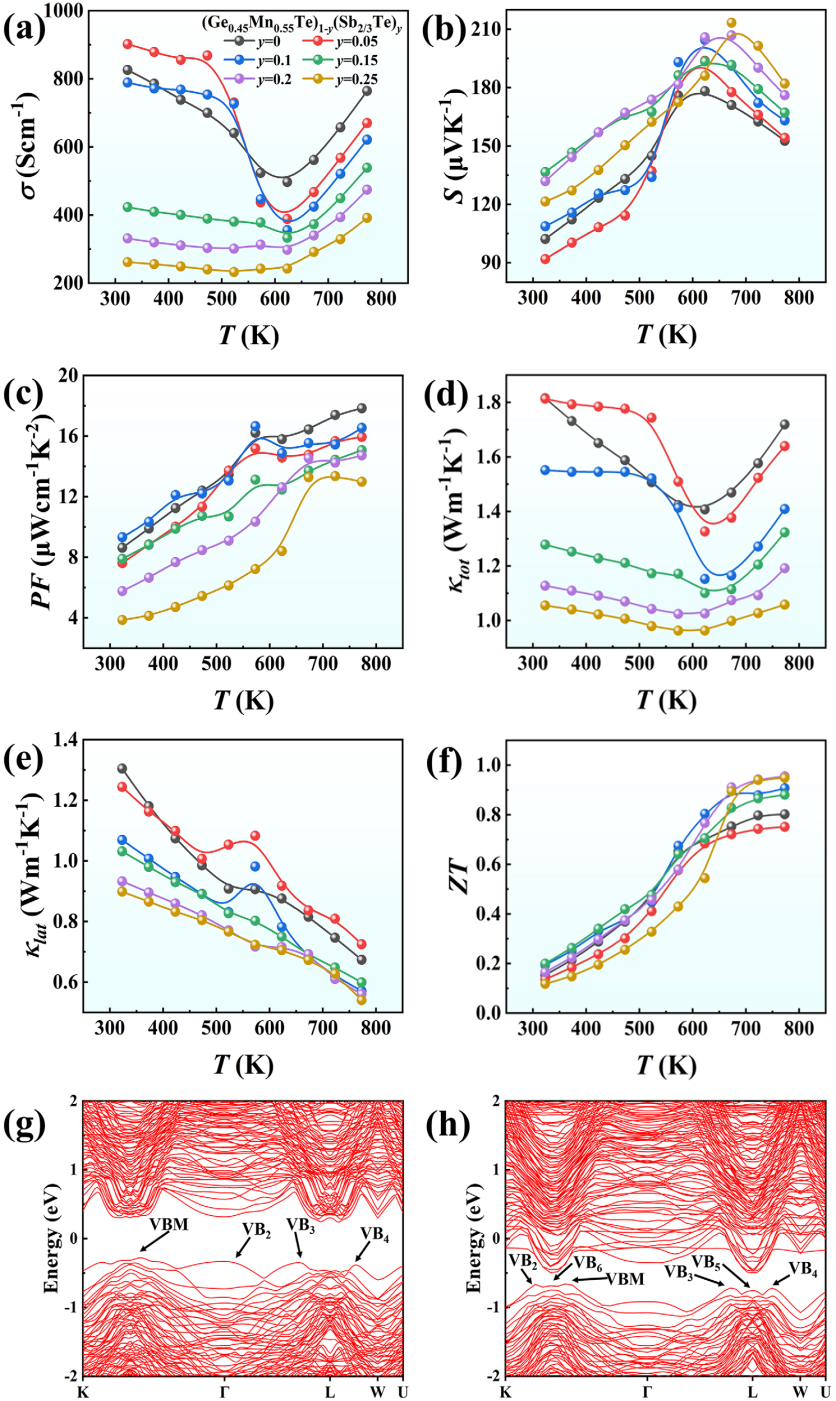
Temperature dependence of a) electrical conductivity *σ*, b) Seebeck coefficient *S*, c) power factor *PF*, d) thermal conductivity *κ_tot_*, e) lattice thermal conductivity *κ_lat_*, and f) figure of merit *ZT* for (Ge_0.45_Mn_0.55_Te)_1‐_ *_y_* (Sb_2/3_Te)*_y_* (*y* = 0, 0.05, 0.1, 0.15, 0.2, and 0.25). Calculated electronic band structures of e) Ge_12_Mn_12_Te_24_ and (f) Ge_10_Mn_10_Sb_4_Te_24_.

Now the optimized Ge_0.45_Mn_0.55_Te is used as a matrix material, and further modulation is realized by alloying different amounts of Sb_2_Te_3_ to the matrix. The synthesized samples with molar ratio *y* of Sb_2_Te_3_ are written as (Ge_0.45_Mn_0.55_Te)_1‐_ *_y_* (Sb_2/3_Te)*_y_* (*y* = 0.05, 0.1, 0.15, 0.20, and 0.25), and their phase structure and lattice constants are shown in Figure S1a,b (Supporting Information). All samples exhibit a single cubic phase structure until a secondary Sb_2_Te_3_ phase emerges when *y* = 0.25. **Figure** **3**a–f shows the thermoelectric properties of (Ge_0.45_Mn_0.55_Te)_1‐_ *_y_* (Sb_2/3_Te)*_y_* (*y* = 0.05, 0.1, 0.15, 0.2, and 0.25) samples. With the increase of *y*, *σ* gradually decreases from 826 to 330 S cm^−1^ at RT; meanwhile, *S* increases significantly. Despite a slight reduction in *PF*, its value still remains at a relatively high level. Upon Sb_2_Te_3_ alloying, the *κ_tot_* value at 323 K decreases from 1.8 W m^−1^ K^−1^ for Ge_0.45_Mn_0.55_Te matrix to 1.13 W m^−1^ K^−1^ for the sample with *y* = 0.2 (Figure 3d). The calculated lattice thermal conductivity *κ_lat_* is shown in Figure 3e. It is obvious that alloying Sb_2_Te_3_ results in a significant and systematic reduction in *κ_lat_*. It is also noted that the *κ_lat_* after Sb_2_Te_3_ alloying exhibits anomaly at ≈500–600 K. Since there is no detectable secondary phase in XRD, we performed SEM and EDS analysis on the *y* = 0.05 sample (Figure S1c,d, Supporting Information) and observed trace amounts of Ge/Mn‐rich precipitations with size of 5–10 µm. We infer that the anomaly of electrical conductivity and electronic thermal conductivity probably results from the dissolution of the Ge/Mn‐rich secondary phase into the matrix at approximately 550 K. Due to the significant reduction in *κ_tot_* and the high level of *PF*, the (Ge_0.45_Mn_0.55_Te)_0.8_(Sb_2/3_Te)_0.2_ sample exhibits a peak *ZT* value of 0.96 at 773 K, which is 20% higher than that of pristine Ge_0.45_Mn_0.55_Te matrix.

To illuminate the underlying reason for increased *ZT* upon Sb_2_Te_3_ alloying, we performed first‐principles density functional theory (DFT) calculations by VASP (Vienna Ab initio Simulation Package) to evaluate the band structure changes.^[^ ^50^, ^51^ ^]^ Details can be found in SI. A special quasi‐random structure (details in Figure S2, Supporting Information) was constructed to serve as the supercell for DFT calculations.^[^ ^52^ ^]^ For simplification and without loss of generality, Figure 3g,h shows the obtained electronic band structures of Ge_12_Mn_12_Te_24_ (= Ge_0.5_Mn_0.5_Te) and Ge_10_Mn_10_Sb_4_Te_24_ (= Ge_0.42_Mn_0.42_Sb_0.17_Te, which is close to (Ge_0.45_Mn_0.55_Te)_0.8_(Sb_2/3_Te)_0.2_≈ Ge_0.36_Mn_0.44_Sb_0.13_Te), respectively. Significant changes in the band structure can be found upon Sb_2_Te_3_ alloying. In addition to the reduction of band gap, more valence band peaks emerge at *K* point to *Г* and *L* points (VB_2_, VB_5_, VB_6_) and the energy difference among these peaks is extremely small, leading to significant enlargement of band degeneracy *N_v_* (projected density of states and partial density of states near Fermi level are shown in Figure S3, Supporting Information). The changes of band structure give rise to remarkable increase in *S*, which compensates the reduction of *σ*, thus, the *PF* value can be maintained at the high level as shown in Figure 3c.

**Figure 4:**
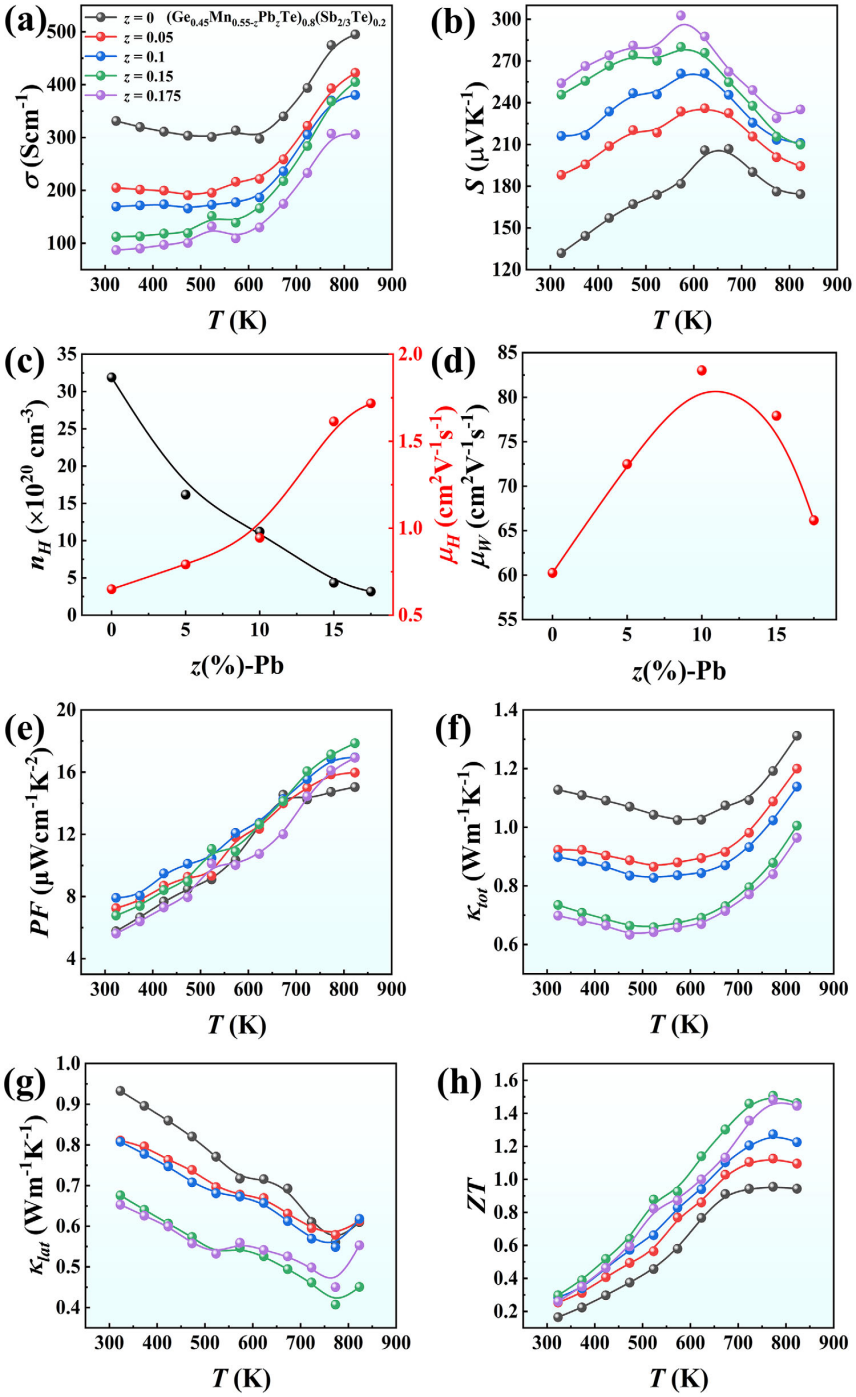
Temperature dependent a) electrical conductivity *σ* and b) Seebeck coefficient *S*. c) Room‐temperature carrier concentration *n_H_* and mobility *µ_H_*, and d) calculated weighted mobility *µ_w_* as a function of Pb content *z*. Temperature dependent e) power factor *PF*, f) total thermal conductivity κ*_tot_*, g) lattice thermal conductivity κ_*lat*_, and h) figure of merit *ZT* for (Ge_0.45_Mn_0.55‐_ *_z_* Pb*_z_* Te)_0.8_(Sb_2/3_Te)_0.2_ (*z* = 0, 0.05, 0.1, 0.15, and 0.175) samples.

Like GeTe, the extremely low cation vacancy formation energy in Ge_0.5_Mn_0.5_Te leads to a very high hole concentration (≈10^21^ cm^−3^).^[^ ^47^ ^]^ Such a high intrinsic carrier concentration typically stands against the advance of its thermoelectric performance. To increase the cation vacancy formation energy for a reduced hole concentration, we use Pb as the substitution element at Mn sites. Through DFT calculations, we find that the cation vacancy formation energies *E*(V_Ge_) of the Pb‐free sample (Ge_12_Mn_12_Te_24_) and Pb‐incorporated sample (e.g., Ge_10_Mn_10_Pb_4_Te_24_) are 0.548 and 4.098 eV, respectively. The significant increase of cation vacancy formation energy is expected to result in obvious hole concentration reduction, which is evidenced by our Hall measurements (**Figure** **4**). Figure S4 (Supporting Information) shows the XRD patterns and lattice constants obtained from (Ge_0.45_Mn_0.55‐_ *_z_* Pb*_z_* Te)_0.8_(Sb_2/3_Te)_0.2_ (*z* = 0, 0.05, 0.1, 0.15, and 0.175) samples.

The measured thermoelectric properties are shown in Figure 4. With the increase of *z*, *σ* decreases gradually (Figure 4a) while *S* behaves in the opposite direction (Figure 4b). Specifically, at 323 K, *σ* drops from 331 S cm^−1^ for *z* = 0 to 87 S cm^−1^ for *z* = 0.175. Correspondingly, *S* rises from 131 to 254 µV K^−1^. Hall measurements at RT (Figure 4c) reveal a significant and systematic reduction in hole concentration alongside a notable improvement in carrier mobility. Furthermore, the weighted mobility *µ_w_*, an important parameter that can quantify the charge carrier transport efficiency, is calculated and presented in Figure 4d.^[^ ^53^ ^]^ It can be seen that the RT *µ_w_* reaches a maximum of ≈83 cm^2^ V^−1^ s^−1^ as Pb content *z* = 0.1, suggesting a significant weakening of charge carrier scattering. Due to the large increase in *S*, the *PF* values of Pb‐incorporated samples are slightly enhanced, *i.e*., a maximum *PF* of 18 µW cm^−1^ K^−2^ is realized at 823 K in the composition of (Ge_0.45_Mn_0.4_Pb_0.15_Te)_0.8_(Sb_2/3_Te)_0.2_ (Figure 4e). The *κ_tot_* is reduced significantly over the entire temperature range from 323 to 823 K; especially, the RT value reduces from 1.13 W m^−1^ K^−1^ for (Ge_0.45_Mn_0.55_Te)_0.8_(Sb_2/3_Te)_0.2_ to 0.73 W m^−1^ K^−1^ for (Ge_0.45_Mn_0.4_Pb_0.15_Te)_0.8_(Sb_2/3_Te)_0.2_, as shown in Figure 4f. Figure 4g represents the calculated *κ_lat_* as a function of *T* for different *z* values; the lowest *κ_tot_* reaches ≈0.4 W m^−1^ K^−1^ at 773 K for the sample with *z* = 0.15, which is close to the amorphous limit.^[^ ^54^, ^55^, ^56^ ^]^ The synergistic optimization of the electrical and thermal transport properties push figure of merit *ZT* to a maximum of 1.5 at 773 K in the composition of (Ge_0.45_Mn_0.4_Pb_0.15_Te)_0.8_(Sb_2/3_Te)_0.2_, which is ≈80% higher than that of the Pb‐free ones (Figure 4h).

**Figure 5:**
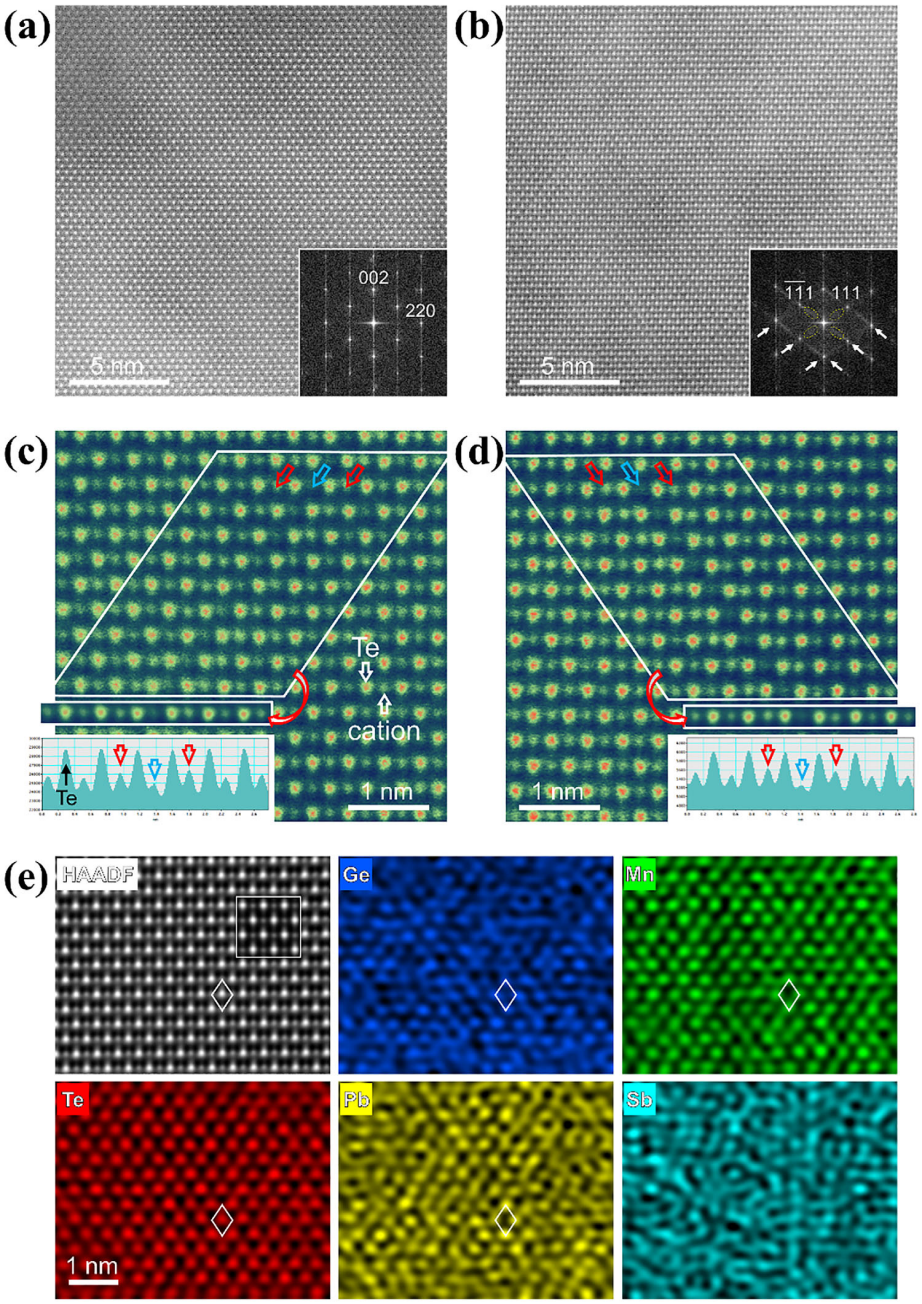
HAADF STEM image and corresponding FFT image (inset) of a) Ge_0.45_Mn_0.55_Te and b) (Ge_0.45_Mn_0.4_Pb_0.15_Te)_0.8_(Sb_2/3_Te)_0.2_. The diffused streak contrast resulting from the presence of localized vdW gaps are indicated by arrows in (b). c,d) Atomic‐resolution HAADF images and the intensity line profiles of the layer‐by‐layer averaged images (outlined by rectangles) obtained from the marked area (by parallelograms). e) Atomic‐resolution HAADF image and corresponding EDS mapping from the (Ge_0.45_Mn_0.4_Pb_0.15_Te)_0.8_(Sb_2/3_Te)_0.2_ matrix. The cation positions are indicated by rhombus. Inset: simulated image.

The microstructure of (Ge_0.45_Mn_0.4_Pb_0.15_Te)_0.8_(Sb_2/3_Te)_0.2_ is investigated using (scanning) transmission electron microscopy ((S)TEM), as shown in **Figure** **5**. First, the cubic structure of (Ge_0.45_Mn_0.4_Pb_0.15_Te)_0.8_(Sb_2/3_Te)_0.2_ matrix grains is confirmed by systematic tilting series of selected area electron diffraction (Figure S5, Supporting Information). At grain boundaries, PbTe and Mn/Ge‐rich precipitates are evident, as shown in Figure S6 (Supporting Information), their size is about 50–100 nm. Besides, high‐magnification high‐angle annular dark‐field (HAADF) STEM images as shown in Figure 5b and Figure S7 (Supporting Information) display the formation of high density localized vdW gaps inside the matrix grains. These vdW gaps are in the {111} habit planes which are close‐packed and result in the diffused streak contrast in the corresponding fast Fourier transfer (FFT) image (indicated by arrows in the inset of Figure 5b). Digital dark‐field image using the selected streak areas (see dashed ellipses) highlights the localized vdW gaps, as shown in Figure S8a (Supporting Information) and the strain components calculated by using geometric phase analysis,^[^ ^57^ ^]^ as shown in Figure S8b–d, reveal that the lattice distortion is confined in the vicinity of localized vdW gaps. In comparison, no such localized microstructure as well as streak contrast is evidenced in the pristine Ge_0.45_Mn_0.55_Te as shown in Figure 5a. Finally, atomic‐resolution HAADF STEM images shown in Figure 5c,d reveal the close‐up configuration of localized vdW gaps in the (Ge_0.45_Mn_0.4_Pb_0.15_Te)_0.8_(Sb_2/3_Te)_0.2_ (≈ Ge_0.36_Mn_0.32_Pb_0.12_Sb_0.13_Te) sample. The bright dots in the grain matrix represent heavier Te atoms while the dark represent (in average) lighter cation atoms dominated by of Ge and Mn. This is further confirmed by image simulation and atomic resolution EDS, as shown in Figure 5e. It should be noted that the atomic number of Sb is next to Te, while its nominal concentration is about 1/10 of the latter. This leads to severe challenge in spatially resolving Sb using spectroscopy against Te. For further analysis, we perform layer‐by‐layer average for the marked regions (white parallelograms) to improve signal‐to‐noise ratio and the resulting images are shown as insets outlined by white rectangles together with the intensity line profiles of atomic columns. It should be also noted that the lateral width of localized vdW gaps is in the range of 10 nm (e.g., see vdW gap 2 in Figure S7, Supporting Information), while the thickness of our TEM sample is about 50–100 nm. This inevitably leads to a situation that the gap overlaps with the grain matrix, thus residual (but low) atomic intensities are still visible, as indicated by blue arrows, which are most likely from the matrix atoms.

The loss of cations in the localized vdW gaps is expected to cause charge imbalance, as we don't see clear intensity changes at the Te columns. To neutralize the local charges, more cations are accumulated to the adjacent columns, as indicated by red arrows. This provides a different mechanism from the case of GeTe,^[^ ^58^, ^59^ ^]^ in which the formation of vdW gaps are correlated with the presence of charged head‐to‐head domains. More gaps and corresponding line profiles can be found in Figure S7 (Supporting Information).

**Figure 6:**
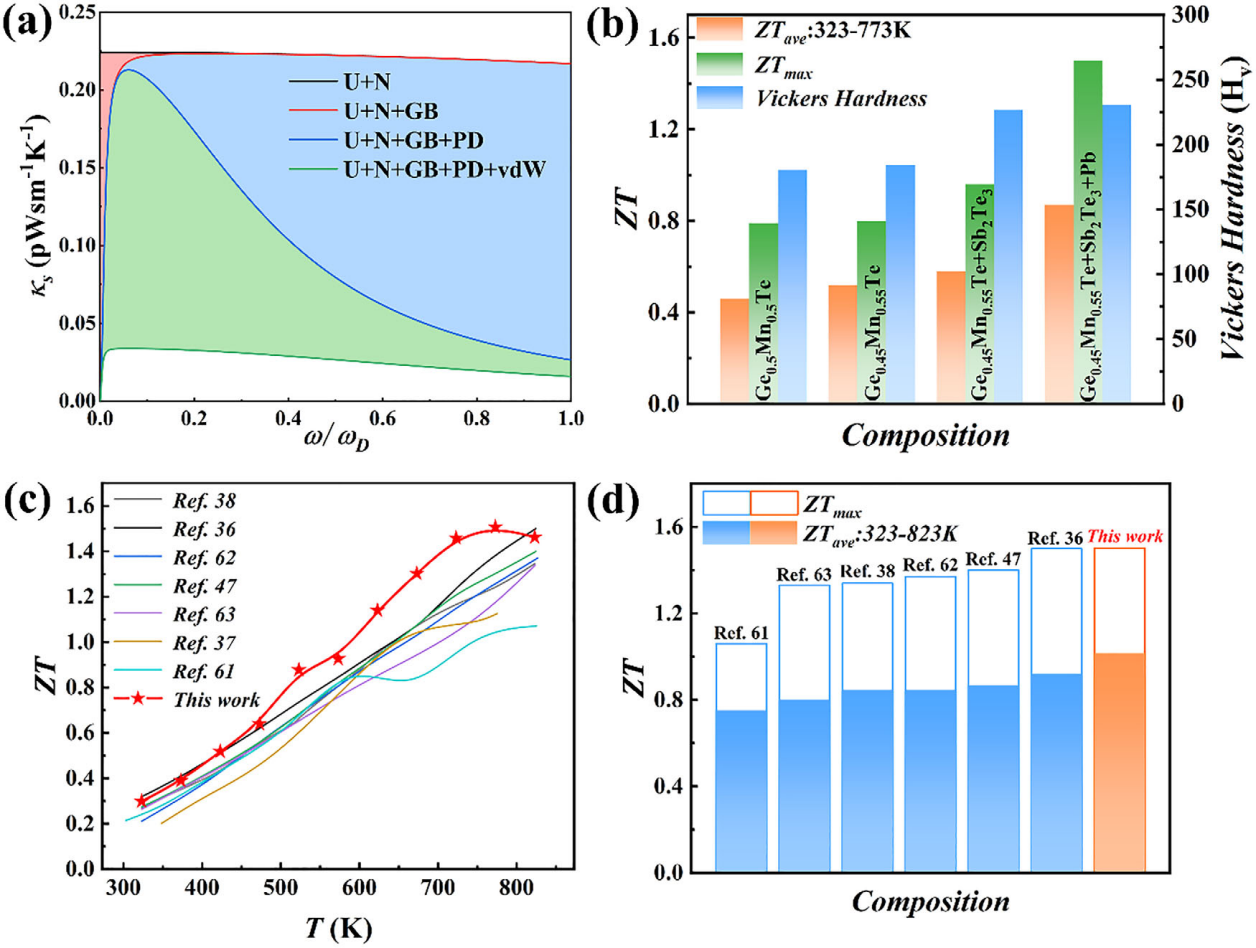
a) Calculated RT spectral lattice thermal conductivity (*κ_s_*) as a function of phonon frequency based on Debye–Callaway model. b) Stepwise enhancements of *ZT_max_*, *ZT_ave_*, and *H_v_* in this work. Comparison of c) temperature‐dependent *ZT* and d) *ZT_max_* and *ZT_ave_* achieved in this work with GeMnTe_2_‐based materials in literatures, where the corresponding compositions are Ge_0.8_Na_0.1_Bi_0.1_MnTe_2_,^[^ ^61^ ^]^ (Sb_2_Te_3_)_0.5_(Ge_0.91_Pb_0.09_Te)_17.5_,^[^ ^37^ ^]^ Ge_0.7_Mn(AgBi)_0.15_Te_2_‐3%Sb,^[^ ^63^ ^]^ Ge_0.9_MnSb_0.1_Te_2_,^[^ ^38^ ^]^ Ge_0.94_Bi_0.06_MnTe_1.94_Se_0.06_,^[^ ^62^ ^]^ Ge_0.92_Pb_0.08_MnTe_2_,^[^ ^47^ ^]^ GeMnTe_2_‐15.1%SbTe.^[^ ^36^ ^]^

To figure out how microstructures affect the phonon transport, we calculate the temperature dependent *κ_lat_* as well as the RT spectral lattice thermal conductivity (*κ_s_*) based on Debye–Callaway model (details in SI) by considering different scattering mechanisms including Umklapp processes (U), normal processes (N), grain boundaries (GB), point defects (PD) and localized vdW gaps. It is seen that our simulated temperature dependent *κ_lat_* agrees quite well with experimental ones (Figure S9, Supporting Information). Moreover, as seen in **Figure** **6**a, the reduction of lattice thermal conductivity at high‐frequency region originates mainly from point defects scattering, considering the fact that evenly distributed Pb/Sb atoms provide strong mass and strain fluctuation as revealed by the aforementioned microstructure characterizations. Meanwhile, abundant localized vdW gaps as revealed in Figure 5 and Figure S7 (Supporting Information) are able to significantly weaken the low‐frequency phonon transport.

So far, we have realized stepwise enhancements of thermoelectric performance based on the cubic‐phase Ge_0.5_Mn_0.5_Te. Specifically, the peak *ZT* (*ZT*_*max*_) increases from 0.8 for Ge_0.5_Mn_0.5_Te to 1.5 for (Ge_0.45_Mn_0.4_Pb_0.15_Te)_0.8_(Sb_2/3_Te)_0.2_, and the average *ZT* (*ZT*_*ave*_) from 0.46 to 0.87 in the temperature range of 323–773 K. We also achieve a significant enhancement of Vicker's hardness *H_v_* (see Figure S10, Supporting Information, for details), i.e., *H_v_* reaches 230.7 in (Ge_0.45_Mn_0.4_Pb_0.15_Te)_0.8_(Sb_2/3_Te)_0.2_, which is 25% higher than the control sample Ge_0.5_Mn_0.5_Te and 72% higher than pristine rhombohedral GeTe.^[^ ^60^ ^]^ The results are summarized in Figure 6b. In Figure 6c,d, we compare the achievement in this work with state‐of‐the‐art Ge_0.5_Mn_0.5_Te‐based thermoelectric materials in literatures,^[^ ^36^, ^37^, ^38^, ^47^, ^61^, ^62^, ^63^ ^]^ the (Ge_0.45_Mn_0.4_Pb_0.15_Te)_0.8_(Sb_2/3_Te)_0.2_ sample exhibits an outstandingly high *ZT_max_* of ≈1.5 at 773 K and remarkably large *ZT_ave_* of ≈0.96 between 323 and 823 K.

## 3 Conclusion

In this work, we successfully construct high‐density localized vdW gaps via Sb_2_Te_3_ alloying with the optimized cubic phase Ge_0.45_Mn_0.55_Te. The localized vdW gaps poses strong scattering on low‐frequency phonons and effectively reduces the *κ_lat_*. Moreover, Sb_2_Te_3_ alloying enlarges the valence band convergence in favor of high *PF*. We also optimize the carrier concentration and improve the weighted mobility by Pb substitution at cation sites, which results in strong phonon scattering in the high‐frequency region. Eventually an ultralow *κ_lat_* down to 0.4 W m^−1^ K^−1^ is achieved at 773 K in (Ge_0.45_Mn_0.4_Pb_0.15_Te)_0.8_(Sb_2/3_Te)_0.2_. This leads to an outstanding peak *ZT* of ≈1.5 at 773 K and a remarkable average *ZT* of ≈0.96 at 323–823 K in the composition of (Ge_0.45_Mn_0.4_Pb_0.15_Te)_0.8_(Sb_2/3_Te)_0.2_, exceeding almost all Ge_0.5_Mn_0.5_Te‐based thermoelectric materials reported so far.

## 4 Experimental Section

The experimental details can be found in the Supporting Information.

## Conflict of Interest

The authors declare no conflict of interest.

## Supporting information



Supporting Information

## Data Availability

The data that support the findings of this study are available from the corresponding author upon reasonable request.
