# Supplementary material for: Constructing Localized Van Der Waals Gaps in Cubic‐Phase GeMnTe2 Thermoelectric Material
Source: Adv Sci (Weinh). 2025 Nov 3;13(4):e17830. doi: 10.1002/advs.202517830 (PMC12822465; doi:10.1002/advs.202517830)
Supplement: Supplementary file 1 — Supporting Information [file ADVS-13-e17830-s001.docx]

Supporting Information

Constructing Localized van der Waals Gaps in Cubic-phase GeMnTe_2_ Thermoelectric Material

Mingrui Zhang, Lingling Wei^*^, Tingting Yang, Weishuai Wang, Fudong Zhang, Mengqi Li, Beiquan Jia, Yalin Shi, Zupei Yang, Rafal E. Dunin-Borkowski, Lei Jin^*^, and Di Wu^*^

**Experimental Section**

**1. Sample Synthesis**

All samples were prepared by high-temperature melting combined with spark plasma sintering (SPS) technology. For the samples of composition Ge_1-_*_x_*Mn*_x_*Te (*x* = 0.5, 0.55, 0.6, 0.7 and 0.8), (Ge_0.45_Mn_0.55_Te)_1-_*_y_*(Sb_2/3_Te)*_y_* (*y* = 0.05, 0.1, 0.15, 0.2 and 0.25), (Ge_0.45_Mn_0.55-_*_z_*Pb*_z_*Te)_0.8_(Sb_2/3_Te)_0.2_ (*z* = 0.05, 0.1, 0.15 and 0.175), high purity Ge (99.99%, Aex), Mn (99.99%, Aladdin), Sb (99.99%, Aladdin), Te (99.99%, Aex), Pb (99.99%, Aladdin) particles were weighted according to stoichiometric ratio and sealed in quartz tube under a vacuum of 10^-4^ Pa. The raw materials were heated to 1373 K in 10 h, soaked at this temperature for 20 h and quenched in water, further annealed at 950 K for 72 h and furnace cooled to room temperature. The obtained ingots were ground into fine powder in an agate mortar, then filled into a graphite mold with diameter of 15 mm and sintered by an SPS system (Sinter Land INC, Japan) at 773 K under axial pressure of 50 MPa for 5 minutes. The obtained pellets were cut into 3 × 3 × 12 mm^3^ cuboids for electrical transport measurements and 6 × 6 × 2 mm^3^ cuboids for thermal expansion coefficient measurements.

**2. Thermoelectric Properties Characterizations**

The Seebeck coefficient and electrical conductivity were measured using a commercial system (ADVANCE RIKO ZEM-3, Japan) in a dilute argon atmosphere, the measurement uncertainty is about 5% for both parameters. Thermal conductivity (*κ_tot_*) was determined using the equation *κ_tot_* = *DρC_p_,* where *D* is the thermal diffusion coefficient, *ρ* is the mass density, and *C_p_* is the specific heat capacity. The thermal diffusion coefficient (*D*) was measured by the laser pulse method (NETZSCH LFA467, Germany) in a nitrogen environment. The uncertainty of thermal conductivity is about 7%. The electronic thermal conductivity (*κ_ele_*) is calculated according to Wiedemann-Franz law: *κ_ele_* = *σLT*, where the Lorenz factor (*L*) can be roughly estimated by the equation: *L* = 1.5 + exp (-|*S*| / 116). Considering the uncertainties of all the parameters, the uncertainty of the calculated *ZT* is about 13%. The sample density was calculated using the Archimedes method, while the specific heat capacity was approximated by the Dulong-Petit limit. Carrier concentration and mobility at room temperature were obtained using the Van der Pauw method with a commercial setup (Lake Shore 8400 Series, USA), with an uncertainty of about 10%.

**3. Phase and Microstructure Characterizations**

Powder X-ray diffraction (XRD) patterns were collected from finely ground samples using an X-ray diffractometer (Rigaku, Japan, Cu-Kα radiation) operating at 40 kV and 20 mA. The 2*θ* scanning range was set from 20° to 80° with a scan rate of 8°/min. Microstructure and elemental distribution at the micron-scale were examined using a scanning electron microscope (SEM, Hitachi SU8020, Japan) equipped with energy-dispersive X-ray spectroscopy (EDS).

Samples for (scanning) transmission electron microscopy investigations were prepared using focused ion beam milling in an FEI Helios NanoLab 460F1 dual beam system. A final thickness of about 50 – 100 nm was achieved. Further thinning often causes lamellar breaking and/or severe structural degradation, thus an ultrathin specimen is hardly prepared. Selected area electron diffraction was performed on ground particles dispersed on holy carbon grid in an FEI Tecnai F20 transmission electron microscope running at 200 kV. Spherical aberration corrected high-angle annular dark-field (HAADF) scanning transmission electron microscopy (STEM) images and atomic-resolution EDS mapping were recorded using a Thermo Fisher Scientific Spectra 30-300 microscope equipped with a high brightness field emission gun, a Super-X energy dispersive X-ray detector. The acceleration voltage is 300 kV. The convergence and collection semi-angles are 25 and 77 – 200 mrad, respectively. Image simulation was performed in Dr. Probe for experimental conditions.^[1]^

**4.** **Mechanical Properties**

To investigate the room temperature mechanical properties, the Vickers microhardness was measured by applying a force of 0.5 kg for 10 s (HV-1000, Shanghai BINGYU, China), and each data was the average value of 5 individual tests.

**5. Density Functional Theory Calculations**

Density functional theory calculations were performed using the projector augmented wave (PAW) method,^[2]^ as implemented in the Vienna Ab initio Simulation Package (VASP),^[3]^ with the Perdew, Burke, and Ernzerhof (PBE) generalized gradient approximation (GGA) utilized for the exchange-correlation energy functional. The DFT+U method was used to describe transition metal Mn with localized d electrons, and the effective interaction parameter *U_eff_* *=* *U* – *J* values were 5 eV. A 2 × 3 × 1 supercell of GeMnTe_2_ (48 atoms) is adopted.^[4]^ The pristine Ge_12_Mn_12_Te_24_ and Sb doped cell (Ge_10_Mn_10_Sb_4_Te_24_) are fully relaxed individually with A 2 × 1 × 4 Monkhorst-Pack k-point mesh and a plane-wave cutoff energy of 500 eV until the forces on atoms were smaller than 1×10^-3^ eV/Å.^[5]^ The energy convergence criterion of 10^-6^ eV were employed for all calculations. In the calculation of density of state (DOS), a denser k-point mesh (4 × 2 × 8) is adopted. And the special quasi-random structure (SQS) approach implemented in the “mcsqs” code of the Alloy Theoretic Automated Toolkit (ATAT) was used to create the supercell, because it can mimic a disordered structure within a small unit cell by reproducing the most relevant pair and multisite correlation functions of a random system.^[6, 7]^ The SQS model is created based on the rock-salt structure (*Fm*$\overline{3}$*m*) in a 48-atom supercell (Ge_12_Mn_12_Te_24_, Ge_10_Mn_10_Sb_4_Te_24_, where Ge, Mn, and Sb are located at cationic sites, Te occupies the anionic site).

For the calculations of vacancy formation energy, we used the following formula:

$E_{\text{f}}=E(\text{defect})-E(\text{pure})-\sum_{\text{i}} n_{\text{i}}\mu_{\text{i}}$ (S1)

*E(defect)* and *E(pure)* are the total energies of unit cells with and without defect separately. *n_i_μ_i_* is the reference energy of *n_i_* added atoms of element *i* with chemical potential *μ_i_*.

**6. Weighted Mobility**

The temperature-dependent weighted mobility (*μ_w_*) was derived from the experimental electrical conductivity *σ* and Seebeck coefficient *S* proposed by G. J. Snyder et al:^[8]^

$\mu_{\text{w}}=331 \frac{\text{cm}^{2}}{\text{Vs}} \left(\frac{\text{m}\Omega\cdot\text{cm}}{\rho}\right) \left(\frac{T}{300 \text{K}}\right)^{-3/2}\left[\frac{\exp\left(\frac{|S|}{k_{\text{B}}/e}-2\right)}{1+\exp\left(-5\left(\frac{|S|}{k_{\text{B}}/e}-1\right)\right)}+\frac{\frac{3}{\pi^{2}} \frac{|S|}{k_{\text{B}}/e}}{1+\exp\left(5\left(\frac{|S|}{k_{\text{B}}/e}-1\right)\right)}\right]$ (S2)

Here, *μ_w_* is the weighted mobility, *ρ* is the electrical resistivity measured in mΩ cm, *T* is the absolute temperature in K, *S* is the Seebeck coefficient, and *k_B_/e* = 86.3 µV K^–1^.

**7. Details of the Debye-Callaway Model**

Using the Debye-Callaway model, the final temperature (*T*)-dependent can be expressed as a sum *κ_lat_* (𝑇) of the spectral lattice thermal conductivity from different frequencies:

$\kappa_{\text{lat}}=\int\kappa_{s}(\omega)d\omega=\frac{1}{3}\int_{0}^{\omega_{a}} C_{s}(\omega)v_{g}(\omega)^{2}\tau_{tot}(\omega)d\omega$ (S3)

Thus, the *κ_s_(ɷ)* is determined by the *C_s_(ɷ)*, the frequency-dependent phonon group velocity *ν_g_ (ɷ)* and total relaxation time *κ_tot_(ɷ)*. Generally, as the phonons in optical branches shows low velocity, only the phonons in acoustic branches are considered to calculate the *κ_lat_*. Thus, the cut-off frequency for acoustic branches *ɷ_a_* is given by $\omega_{a}=\left(\frac{6\pi^{2}}{V_{\text{cell}}}\right)^{1/3}$, $v_{s}=\left(\frac{6\pi^{2}}{NV_{\text{av}}}\right)^{1/3}$, where *N*, *V_av_* and *v_s_* the atomic numbers in a primitive cell, average atomic volume and sound speed respectively. For simple approximation, the frequency-dependent phonon group velocity *ν_g_ (ɷ)* is set as a constant value *𝑣_s_*, and *𝜅_lat_* is calculated by the following equation:

$\kappa_{\text{lat}}=\frac{k_{B}}{2\pi^{2}v_{s}}\left(\frac{k_{B}}{h}\right)\int_{0}^{\theta_{D}/T} \tau_{tot}(x)\frac{x^{4}e^{x}}{(e^{x}-1)^{2}}dx$ (S4)

(S5)

The dimensionless variable *x* in equation is defined as , where *ω* is the phonon frequency. The *τ_tot_ (x)* is the reciprocal sum of the relaxation-times from different scattering mechanisms according to the Matthiessen’s rule:

$\tau=\tau_{U}^{-1}+\tau_{N}^{-1}+\tau_{B}^{-1}+\tau_{PD}^{-1}+\tau_{vdW}^{-1}$ (S6)

Where *τ_U_*^-1^, *τ_N_*^-1^, *τ_B_*^-1^, *τ_PD_*^-1^ and *τ_vdW_*^-1^ are the contributions from the Umklapp phonon-phonon scattering, normal phonon-phonon scattering, boundary scattering, point-defect scattering and localized van der Waals gaps scattering.

The *τ_U_*^-1^ is calculated from the following equation:

(S7)

Where *𝛾* and *M_av_* are the Gruneisen parameter, and the atomic mass, respectively.

The *τ_N_*^-1^ is calculated from the following equation:

$\tau_{N}^{-1}=B_{N}\tau_{U}^{-1}$ (S8)

Where *B_N_* is the ratio of normal phonon scattering to Umklapp scattering.

The *τ_B_*^-1^ is calculated from the following equation:

$\tau_{B}^{-1}=\frac{v_{s}}{D}$ (S9)

Where *v_s_* is the average sound velocity and *D* is the experimentally determined grain size.

The *τ_PD_*^-1^ is calculated from the following equation:

$\tau_{PD}^{-1}=\frac{V_{av}\Gamma}{4\pi v_{s}^{3}}\omega^{4}=\frac{V_{av}\Gamma}{4\pi v_{s}^{3}}(\frac{k_{B}T}{h})^{4}x^{4}$ (S10)

Where *Г* is the disorder scattering parameter.

The *τ_vdW_*^-1^ is calculated from the following equation:

$\tau_{vdW}^{-1}=0.7\frac{a_{lat}^{2}\gamma^{2}N_{s}}{v_{s}^{2}\omega^{2}}$ (S11)

Where *a_lat_*, *γ*, *N_s_*, *ν_s_* and *ω* are the average lattice parameter, Grüneisen parameter, density of gaps, sound speed and frequency, respectively.

Detailed parameters are shown in **Table S1**.^[9-11]^

**Table S1.** Parameters of Debye-Callaway model

| **Parameter** | **Symbol** | **Values** | **Source** |
| --- | --- | --- | --- |
| Average atomic mass/kg | *M_av_* | 1.764*10^-25^ | experiments |
| Average atomic mass volume/kg | *V_av_* | 2.554*10^-29^ | experiments |
| Grüneisen parameter | *γ* | 1.4833 | Ref.11 |
| Average sound velocity (m/s) | *v_s_* | 2362 | Ref.9 |
| Longitudinal velocity (m/s) | *v_L_* | 3670 | Ref.9 |
| Transverse velocity (m/s) | *v_T_* | 2129 | Ref.9 |
| Debye temperature (K) | *Ɵ_a_* | 239 | experiments |
| Umklapp to normal ratio | *B_N_* | 3.5 | fitted |
| Characteristic of the vibrational | *b* | 0.9 | fitted |
| Grain size (m) | *D* | 3*10^-5^ | experiments |
| Disorder scattering parameter | *Г* | 0.1 | fitted |
| Density of gaps (m^-2^) | *N_s_* | 3.25*10^7^ | fitted |
| Lattice constant | *a_lat_* | 5.89*10^-10^ | experiments |

**Supporting Figures:**

**
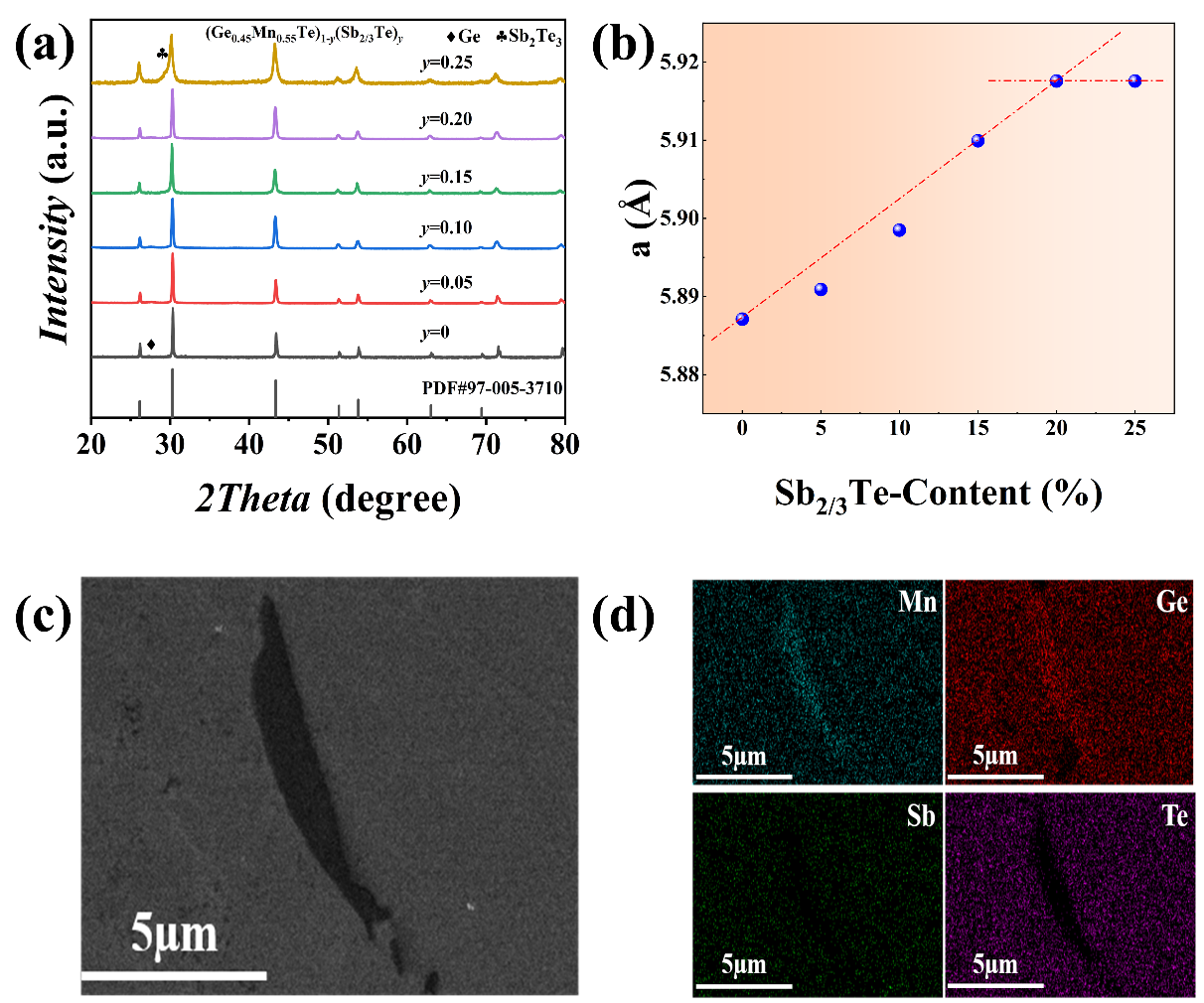
**

**Figure S1.** (a) Room temperature powder XRD diffraction pattern of (Ge_0.45_Mn_0.55_Te)_1-_*_y_*(Sb_2/3_Te)*_y_* (*y* = 0.05, 0.1, 0.15, 0.2 and 0.25), (b) measured lattice constants, (c) micron-scale precipitate and (d) EDS patterns in the sample with *y* = 0.05.


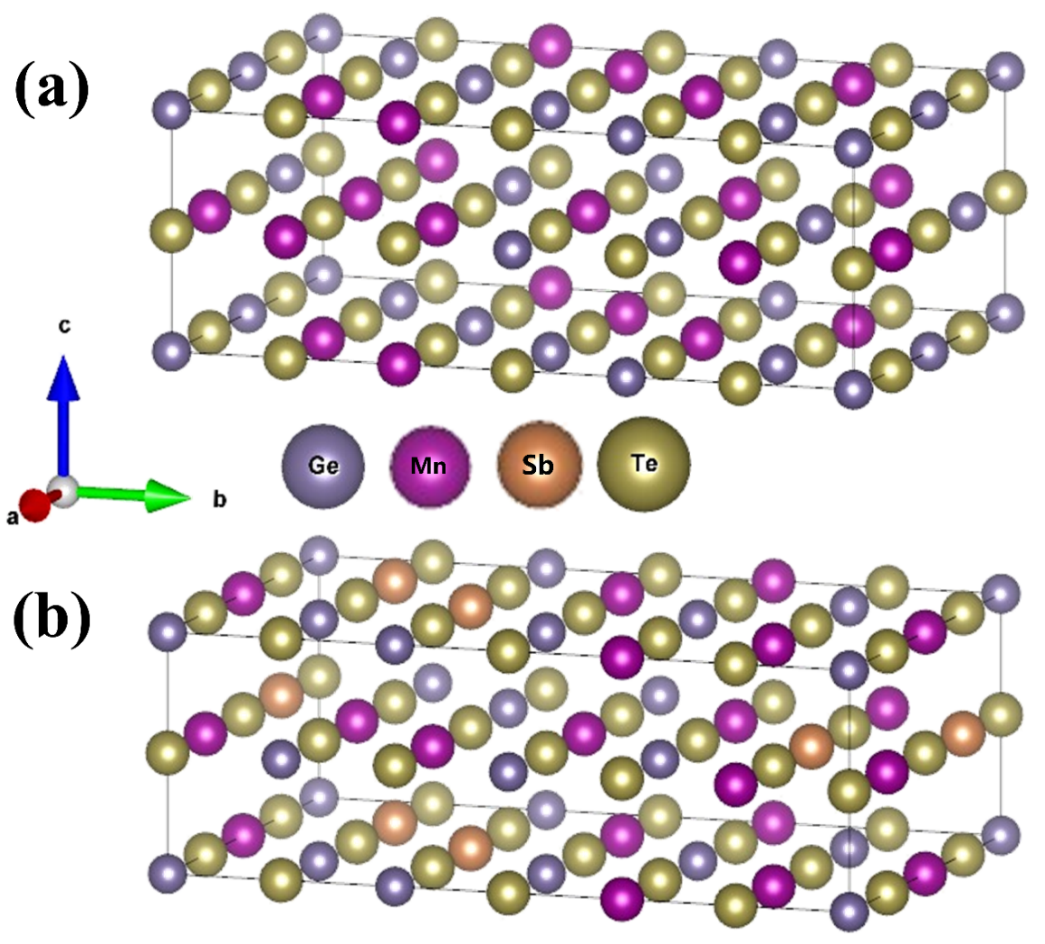


**Figure S2.** Detailed special quasi-random structure of (a) Ge_12_Mn_12_Te_24_ and (b) Ge_10_Mn_10_Sb_4_Te_24_ used for DFT calculations.


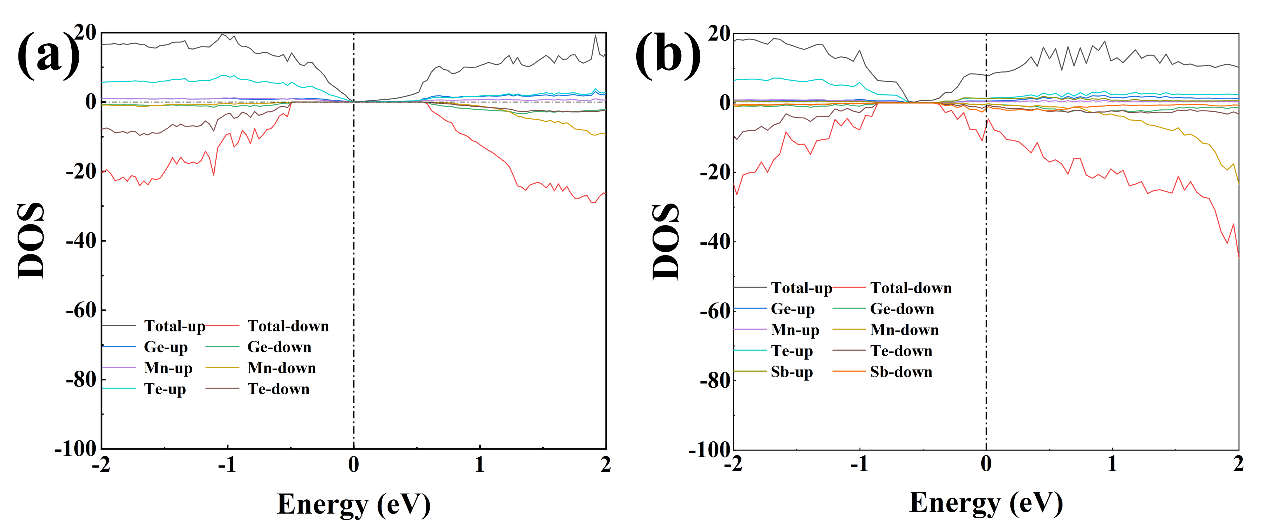


**Figure S3.** Density of state (DOS) and Partial density of state (PDOS) for (a) Ge_12_Mn_12_Te_24_ and (b) Ge_10_Mn_10_Sb_4_Te_24_.


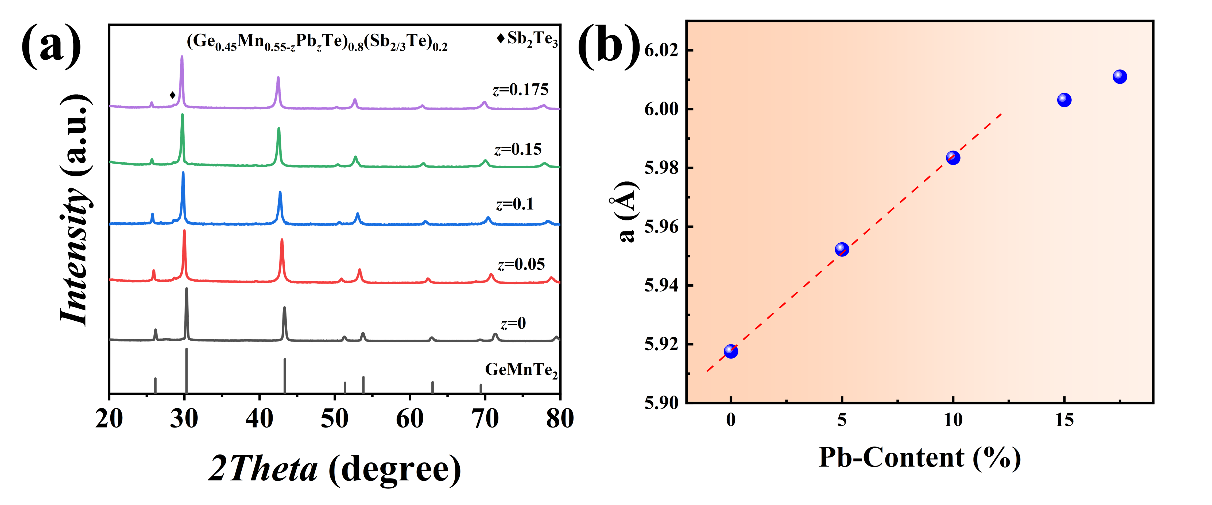


**Figure S4.** (a) Room-temperature powder XRD diffraction pattern of (Ge_0.45_Mn_0.55-_*_z_*Pb*_z_*Te)_0.8_(Sb_2/3_Te)_0.2_ (*z* = 0, 0.05, 0.1, 0.15 and 0.175), and (b) calculated lattice constants.


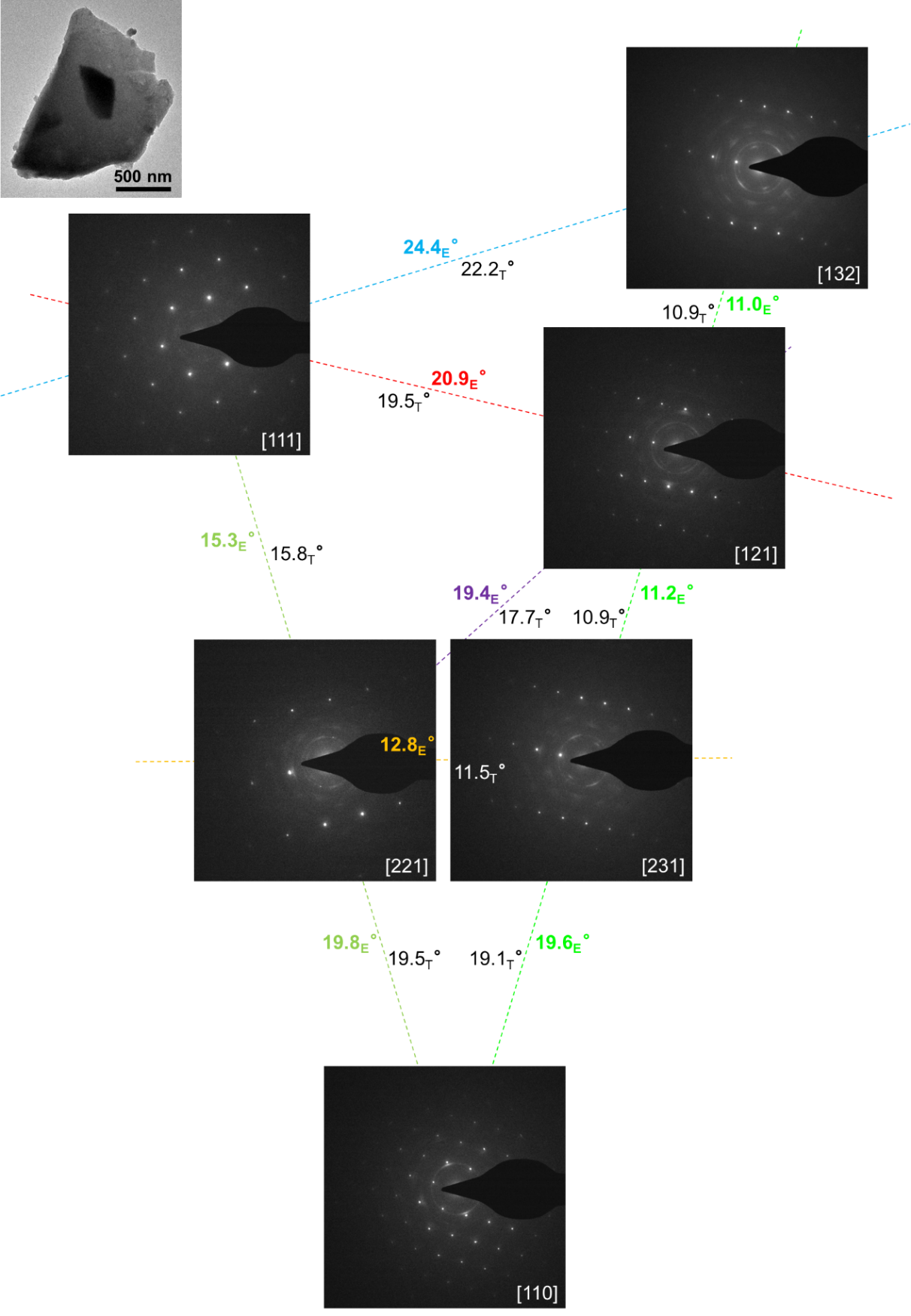


**Figure S5.** Systematic tilting series of selected area electron diffraction revealing the cubic phase structure of (Ge_0.45_Mn_0.4_Pb_0.15_Te)_0.8_(Sb_2/3_Te)_0.2_. Values with subscripts E and T denote the experimentally measured and theoretically calculated angles between the adjacent directions.


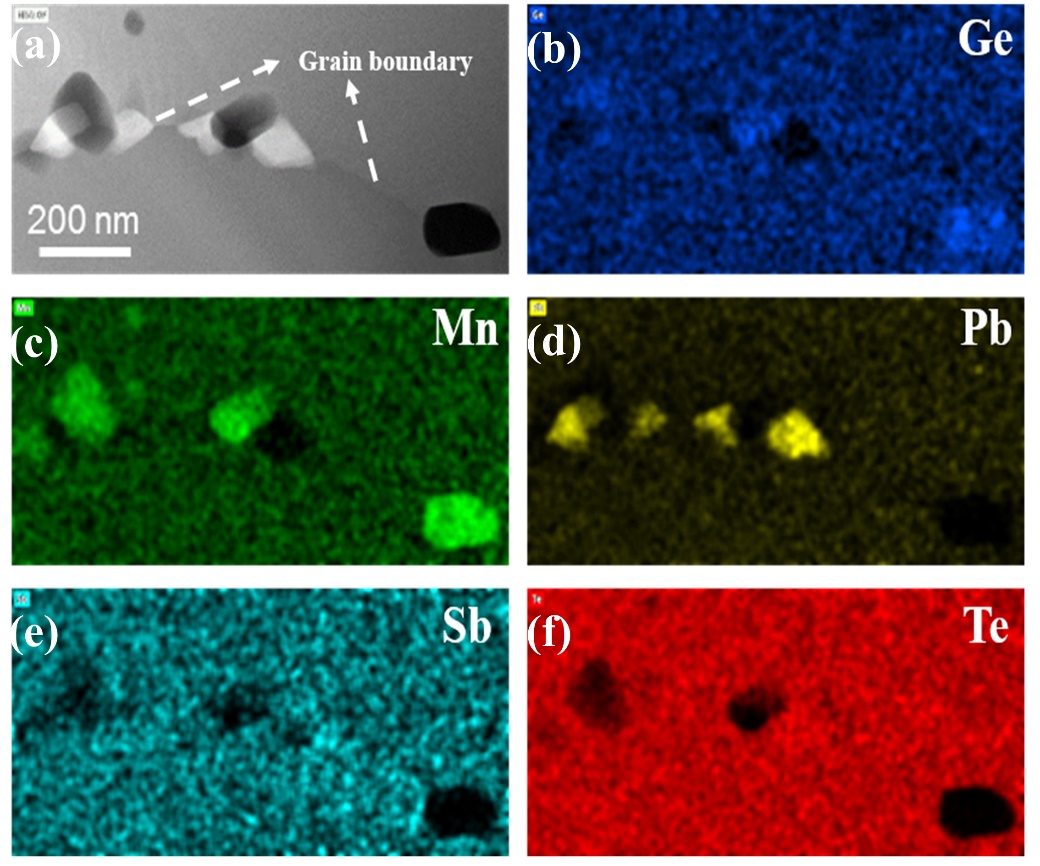


**Figure S6.** (a) Low-magnification HAADF STEM image and (b)-(f) corresponding EDS maps showing PbTe and Ge/Mn-rich precipitates in the (Ge_0.45_Mn_0.4_Pb_0.15_Te)_0.8_(Sb_2/3_Te)_0.2_ sample.


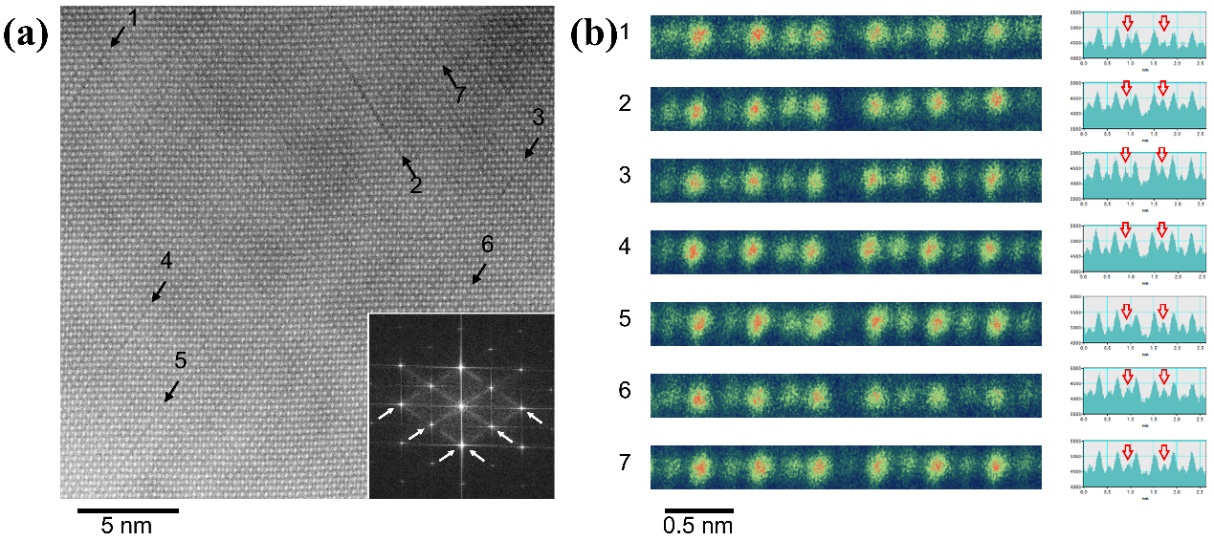


**Figure S7.** (a) HAADF STEM image and corresponding FFT (inset) showing the presence of localized vdW gaps in (Ge_0.45_Mn_0.4_Pb_0.15_Te)_0.8_(Sb_2/3_Te)_0.2_. (b) Layer-by-layer averaged images of localized vdW gaps indicated by 1 – 7 in (a) and corresponding intensity line profiles of the atomic columns across the vdW gaps. Red arrows mark cation columns with higher intensity.


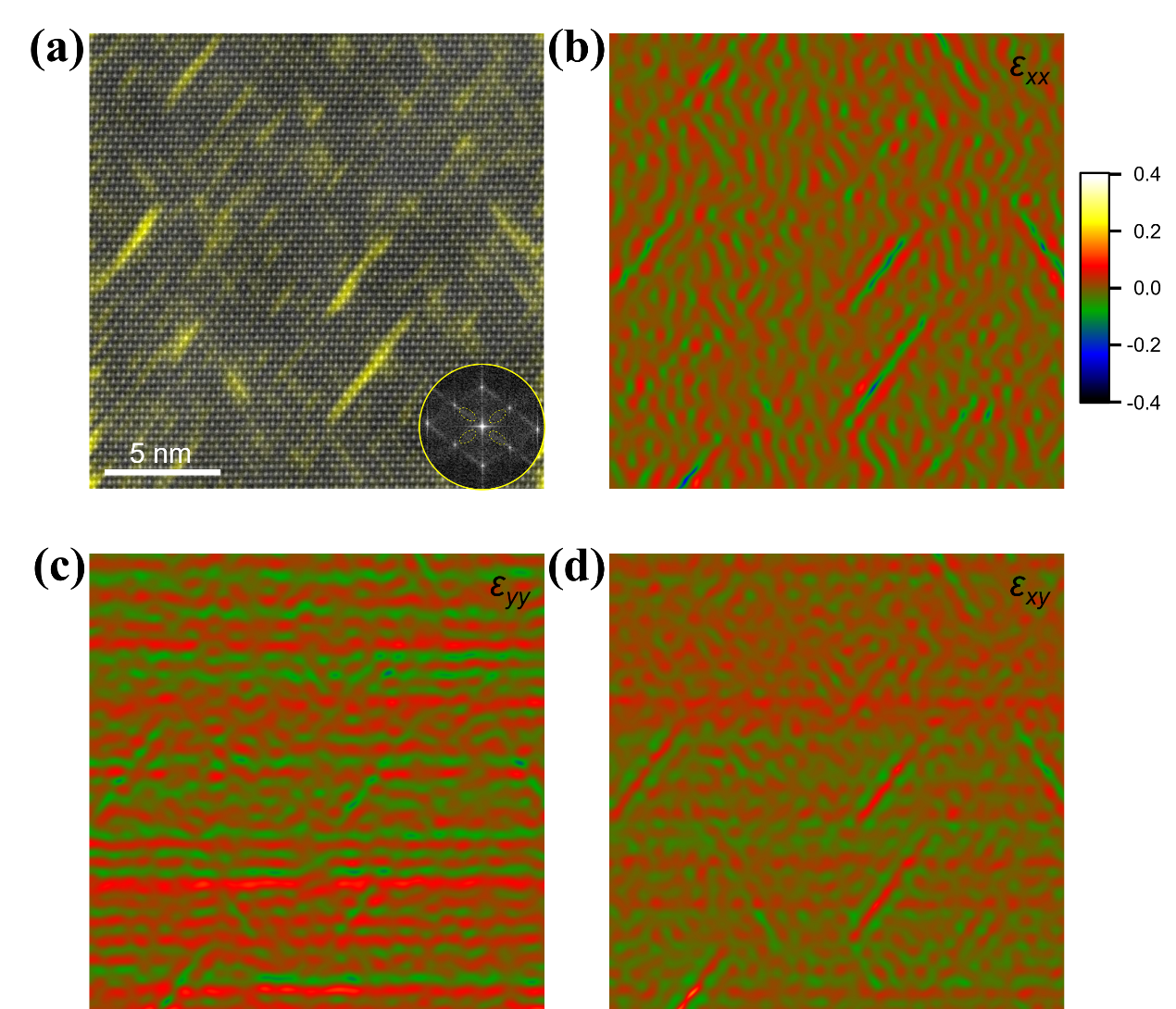


**Figure S8.** (a) Digital dark-field image (*i.e.*, inverse Fourier transform) generated by using the selected streak areas (see dashed ellipses) highlights the localized vdW gaps. (b) to (d) Strain components of *ε_xx_*, *ε_yy_* and *ε_xy_*, respectively, calculated by using geometric phase analysis.^[12]^


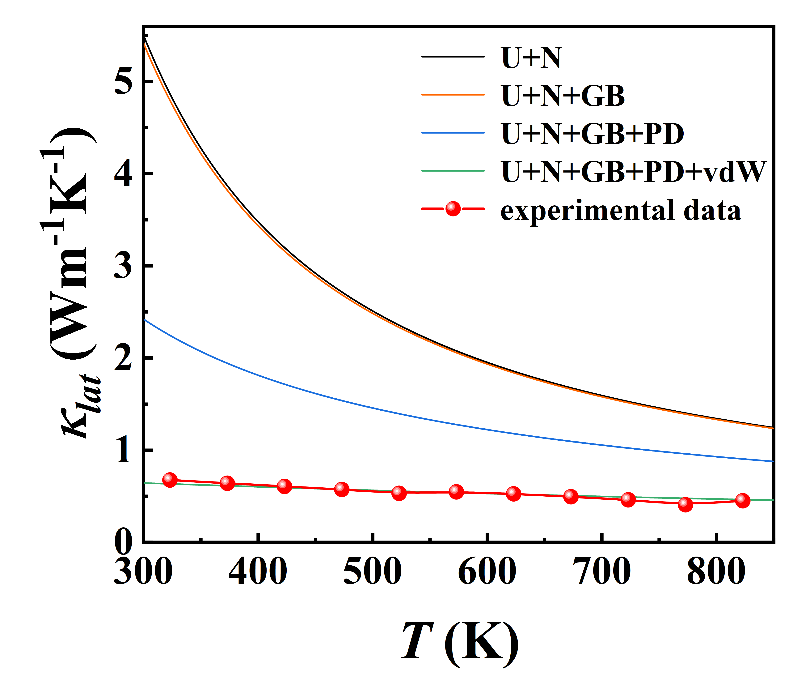


**Figure S9.** Fitting of temperature-dependent lattice thermal conductivity *κ_lat_* of the (Ge_0.45_Mn_0.4_Pb_0.15_Te)_0.8_(Sb_2/3_Te)_0.2_ sample using Debye-Callaway model by considering Umklapp phonon-phonon scattering (U), normal phonon-phonon scattering (N), boundary scattering (B), point defect scattering (PD) and van der Waals gaps scattering (vdW) processes.


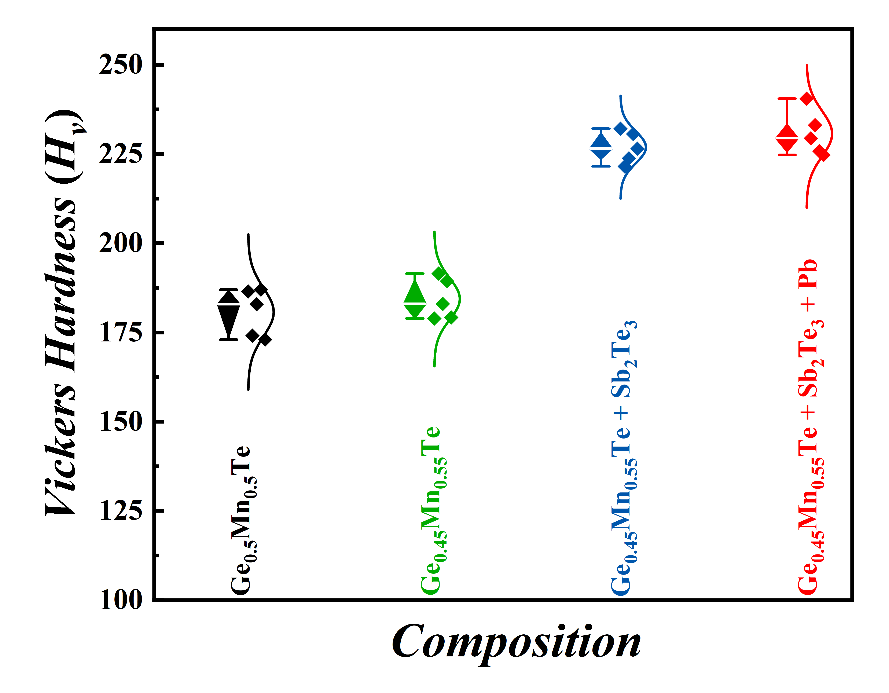


**Figure S10.** Stepwise enhancements of Vickers hardness (*H_v_*) in this work.

**References**

[1] J. Barthel, “Dr. Probe: A software for high-resolution STEM image simulation,” *Ultramicroscopy* **2018**, *193*, 1-11. https://doi.org/10.1016/j.ultramic.2018.06.003.

[2] P. E. Blöchl, “Projector augmented-wave method,” *Phys. Rev. B* **1994**, *50*, 17953-17979. https://doi.org/10.1103/PhysRevB.50.17953.

[3] V. Wang, N. Xu, J.-C. Liu, G. Tang, W.-T. Geng, “VASPKIT: A user-friendly interface facilitating high-throughput computing and analysis using VASP code,” *Comput. Phys. Commun.* **2021**, *267*, 108033. https://doi.org/10.1016/j.cpc.2021.108033.

[4] M. Zou, H. Dong, Y. Lu, T. Wang, D. Su, Y. Wang, X. Pan, Y. Zou, “Determining the formation enthalpies and phase diagram of high-density uranium fuels by mixing GGA and GGA + U calculations,” *Comput. Mater. Sci.* **2025**, *249*, 113626. https://doi.org/10.1016/j.commatsci.2024.113626.

[5] H. J. Monkhorst, J. D. Pack, “Special points for Brillouin-zone integrations,” *Phys. Rev. B* **1976**, *13*, 5188-5192. https://doi.org/10.1103/PhysRevB.13.5188.

[6] G. Lakits, A. Arnau, H. Winter, “Slow-particle-induced kinetic electron emission from a clean metal surface: A comparison for neutral and ionized projectiles,” *Phys. Rev. B* **1990**, *42*, 15-24. https://doi.org/10.1103/PhysRevB.42.15.

[7] A. van de Walle, P. Tiwary, M. de Jong, D. L. Olmsted, M. Asta, A. Dick, D. Shin, Y. Wang, L.-Q. Chen, Z.-K. Liu, “Efficient stochastic generation of special quasirandom structures,” *Calphad* **2013**, *42*, 13-18. https://doi.org/10.1016/j.calphad.2013.06.006.

[8] G. J. Snyder, A. H. Snyder, M. Wood, R. Gurunathan, B. H. Snyder, C. Niu, “Weighted Mobility,” *Adv. Mater.* **2020**, *32*, 2001537. https://doi.org/10.1002/adma.202001537.

[9] Q. Mei, C. Xie, J. Liu, Y. Wang, J. Cui, L. Liao, C. Liao, W. Xu, S. Li, Q. Zhang, X. Tang, G. Tan, “Unveiling the Role of Nontrivial Electronic Structure and Lattice Softening in the Excellent Thermoelectric Performance of MnGeTe_2_ Alloys near the Ioffe–Regel Limit,” *Adv. Energy Mater.* **2025**, *15*, 2500937. https://doi.org/10.1002/aenm.202500937.

[10] M. Caro, A. Tamm, A. A. Correa, A. Caro, “Role of electrons in collision cascades in solids. I. Dissipative model,” *Phys. Rev. B* **2019**, *99*, 174301. https://doi.org/10.1103/PhysRevB.99.174301.

[11] J. Dong, Y. Jiang, Y. Sun, J. Liu, J. Pei, W. Li, X. Y. Tan, L. Hu, N. Jia, B. Xu, Q. Li, J.-F. Li, Q. Yan, M. G. Kanatzidis, “Discordant Distortion in Cubic GeMnTe_2_ and High Thermoelectric Properties of GeMnTe_2_-*x*%SbTe. *J. Am. Chem. Soc.* **2023**, *145*, 1988-1996. https://doi.org/10.1021/jacs.2c12877.

[12] M. J. Hÿtch, E. Snoeck, R. Kilaas, “Quantitative measurement of displacement and strain fields from HREM micrographs,” *Ultramicroscopy* **1998**, *74*, 131-146. https://doi.org/10.1016/S0304-3991(98)00035-7.
